# Underlying Principles of a Covid-19 Behavioral Vaccine for a Sustainable Cultural Change

**DOI:** 10.3390/ijerph17239066

**Published:** 2020-12-04

**Authors:** Kalliu Carvalho Couto, Flora Moura Lorenzo, Marco Tagliabue, Marcelo Borges Henriques, Roberta Freitas Lemos

**Affiliations:** 1 Department of Behavioural Sciences, Faculty of Health Sciences, OsloMet—Oslo Metropolitan University, 0167 Oslo, Norway; marco.tagliabue@oslomet.no; 2 Department of Basic Psychological Processes, Psychology Institute, Campus Universitário Darcy Ribeiro, University of Brasília, Brasília 70910-900, Brazil; flora.lorenzo@gmail.com; 3 Federal University of Jataí, Jataí 75801-615, Brazil; marcelobhenriques@ufg.br; 4 Fralin Biomedical Research Institute at Virginia Tech Carilion, Roanoke, VA 24016, USA; rflemos@vtc.vt.edu

**Keywords:** behavioral vaccine, COVID-19, cultural change, policymaking, metacontingency, behavior analysis

## Abstract

Until pharmacological measures are effective at containing the COVID-19 outbreak, adopting protective behaviors is paramount. In this work, we aim at informing interventions to limit the spread of the contagion and prepare against any future outbreaks by developing a behavioral framework to interpret and prescribe both the individual and large-scale uptake of non-pharmaceutical measures. First, we analyze the barriers and facilitators to adherence to protective behaviors according to a three-term contingency by exploring potential gaps in terms of setting stimuli, motivating operations, delayed consequences, and positive or negative consequences. We explore their roles in the likelihood of individual compliance to protective behaviors, taking physical distancing as an example of functional analysis. Second, we interpret contagion control as the cumulative effect of large-scale adherence to protective behaviors. We explore the interrelations between societal problems caused or amplified by similar behaviors presented by many individuals and the coordination of agents or agencies aiming at promoting large-scale behavioral change. Then, we highlight the potential of developing a behavioral vaccine, and practical steps for applying it to promote sustainable cultural change that may protect against health, social, and economic losses in future outbreaks.

## 1. Introduction

The extent of the COVID-19 outbreak is unprecedented in contemporary times. On 11 March 2020, the WHO declared COVID-19 a pandemic, and more than 1.48 million deaths worldwide have been registered as of December 2, 2020 [1]. However, it is difficult to measure the health, social, and economic impacts associated with the COVID-19 outbreak due to the novelty of the virus and its interaction with the current social and geopolitical scenario. Compartmental and statistical epidemiological models are amongst the most frequently used approaches to predict the COVID-19 case fatality rate (CFR) and reproduction rate (R_0_), which represent crucial measures for estimating the progression and outcomes of the disease [2,3,4,5]. The CFR is represented by the proportion of deaths relative to the confirmed cases, and the R_0_ represents the spread potential of a virus within a population. For example, with an R_0_ = 2, an infected person will transmit it to an average of two other individuals. Therefore, in order to contain the disease outbreak, it is important to achieve an R_0_ < 1, which will produce a downtrend in transmission, possibly eradicating the illness.

The CFR of COVID-19 has been estimated with different magnitudes from region to region [6] and is influenced by interactions between variables, such as the characteristics of the virus (e.g., lethality), the characteristics of the population (e.g., comorbidities and age), and the infrastructure (e.g., health care system capacity). CRF estimates may also be influenced by failures in surveillance and notification systems, leading to an underestimation of the incidence and mortality of a disease [7]. Even though R_0_ rates do not affect CFR directly, high values of R_0_ may lead to high death rates due to a low capacity to formulate early diagnosis and provide proper treatment. Nevertheless, a lack of medical resources may also lead to the unnecessary death of patients with other diagnoses due to an overloaded health care system [8,9].

Although vaccination is among the most effective interventions to keep values of R_0_ < 1, there is a delay between a new infectious disease outbreak and the development of vaccines and other effective pharmacological containment measures. There is also a delay between the development of vaccines and their effectiveness in promoting heard immunity. Therefore, alternative measures to reduce R_0_ may take at least two complementary directions: (i) intensive testing and contact tracing, and (ii) population-wide practices of protective behaviors, such as self-hygiene, physical distancing, self-quarantine, the avoidance of mass congregations, and the use of personal protective equipment [10]. If adopted in the early phases of an outbreak, behavioral measures may prevent infectious diseases from reaching pandemic levels. These measures are often aimed at mitigating impacts on affected areas and preventing, or at least delaying, exponential spread in regions where cases are under control.

Modern computational and mathematical models highlight the importance of considering behavioral variables when predicting the spread of infectious diseases. For example, Kim et al. [11] modelled the COVID-19 outbreak in the Republic of Korea, showing reductions in R_0_ as a function of the adoption of protective behaviors. Chowell et al. [12] found that the adoption of protective behaviors also decreased CRF values, especially when mass testing was not possible. Although transmission and mortality rates differ greatly across territories [6], one could argue that sharing a common risk of infection and death threat would lead most citizens to adhere to experts’ recommendations. However, individuals’ habits and choices are shaped and maintained differently by a wide range of environmental variables. Therefore, behavior analysis may provide a useful framework to understand how environmental events influence and are influenced by human behavior.

The aim of this study is twofold. First, we aim to describe environmental conditions that may decrease or increase the likelihood of individuals engaging in protective behaviors. The adoption of these behaviors serves as an “immunization” against contracting and spreading the COVID-19 virus. In order to offer this general analysis, we first present basic principles of learning and their usefulness in interpreting some of the hindrances and facilitators to the adherence to protective behaviors. We provide a functional analysis of physical distancing as an example of targeted protective behavior. Furthermore, we describe how principles of learning may be used to inform the development of a behavioral vaccine.

The second aim of this paper is to analyze how organized efforts across sectors of a society may be arranged, allowing for the implementation of a behavioral vaccine. Implementing a behavioral vaccine should be timely and consider the outbreak status in each region; furthermore, different sectors of society must “act” in a coordinated fashion We advance a framework resting on culturo-behavior science to highlight the importance of developing a nationwide system response that may lead to societal protection in current and future outbreaks.

## 2. The Underlying Learning Principles of a Behavioral Vaccine

Experimental and applied studies have shown two functionally distinct environmental variables that influence behavior: antecedents and consequences [13]. Antecedents are contextual events that signal the likelihood of reinforcing and aversive consequences for the emissions of a response. That is, antecedents are cues to the type of consequence that will follow a response in each circumstance. Consequences are environmental events that affect behavioral classes, increasing or decreasing the probability of future occurrences. The functional relationship between a behavioral class, antecedents, and consequences is termed contingency. Changes in antecedents and consequences produce well-researched and systematic effects on behavior, which are described in terms of behavioral principles, e.g., [14,15]. The outcomes of organism–environment interactions are considered behavioral principles insofar as there is a regularity across a wide range of species [16].

**Figure 1:**
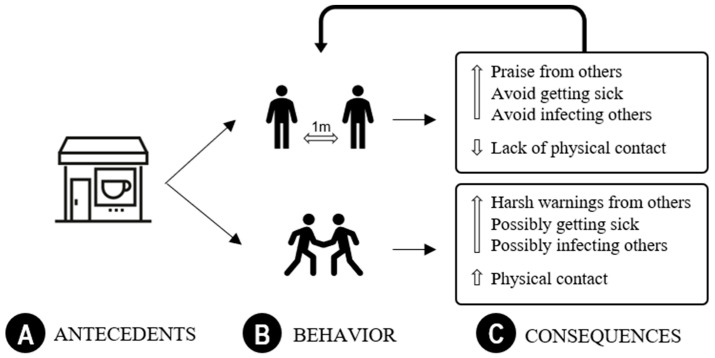
Three-term contingency description of the behavior of greeting a friend keeping physical distance or proximity.

The first step to design a successful intervention is to carefully identify target behaviors. Knowing that behavior is affected by its relationship with antecedents and consequences, the second step is to describe the interactions between antecedent, behavior, and consequences. The environment where behavior takes place is a context containing antecedents that signal that the emission of a certain behavioral class may be followed by a set of consequences. Figure 1 depicts an example where meeting an old friend in a coffee shop (A) may be a context for shaking hands or saying “Hi!” from a 1 m distance (B). The consequences (C) of each behavioral class will increase or decrease their probability of occurring in future occasions.

Embry [17,18] proposed that the power, cost-effect, and predictability of interventions at the population level can be achieved by adopting simple behavior strategies that focus on the antecedents and consequences of behaviors and that impact the societal level (e.g., drivers’ seatbelt use and handwashing among medical personnel). These results can be achieved by adopting routines and practices that have been scientifically linked to reduce morbidity and mortality, thus functioning as a behavioral vaccine. In order to implement behavioral vaccines at the population level, the stakeholders must first know and understand the variables and principles influencing individual engagement in protective measures. In the next section, we describe the components of behavioral contingency (antecedent-behavior-consequence) and ways to take them into consideration when developing a behavioral vaccine. The elements described below were chosen due to their illustrative value for the phenomena analyzed. However, there is a vast range of behavior analytic concepts that could be equally useful but that were not considered due to the constraints of this work.

### 2.1. Targeting Behaviors Through a Clear Operational Definition

International guidelines for protective behaviors may appear to be descriptive enough. However, the interpretation of their form and when they should be adopted may vary. A precise operational definition of target behaviors is the first important step before identifying the variables that affect them. It may consider a behavioral class in terms of topography, temporality, frequency, and effort. Below, we describe important properties of an operational definition using different protective behaviors as examples. It is important to note that each property described is functionally dependent on the other two components of a contingency. That is, topography, temporality, frequency, and effort are contextually dependent (antecedents) and influenced by their effect (consequences).

#### 2.1.1. Topography

Protective behaviors recommended by the authorities may vary in terms of their form and from context to context. For example, social and physical distancing have been topographically defined as “keeping space between yourself and other people outside of your home” [19]. This measure comprises a collection of behaviors aimed at decreasing R_0_ values by diminishing the probability of interpersonal contact transmission [20]. However, for highly transmissible diseases, precise definitions are more effective as guidance for effective protection. It is preferable to specify the exact distance that should be kept between individuals in different situations. For instance, the Center for Disease Control and Prevention (CDC) recommends keeping at least 2 m distance between one another [19]. In special cases, it was suggested that distancing for runners and cyclists should be extended to 5–10 m due to the high volatility of micro-droplets [21]. Social containment encompasses limiting gathering in crowds to avoid situations in which the minimum interpersonal distance cannot be enforced. The topography to be prioritized depends on contextual features and the stage of the pandemic.

#### 2.1.2. Temporality

Still considering physical distancing as an example of target behavior, other formulations have proposed temporal distancing as an alternative [22]. Use of time can be managed to avoid crowds—for example, by scheduling alternative workhours and workstations for company employees, restricting access to stores and services, and discouraging the use of public transport whenever possible. In this way, the number of employees at the workplace at once is smaller, facilitating physical distancing. The implementation of working schedules may also facilitate manual and digital contact tracing, which has been suggested to be an efficient protective measure [23].

#### 2.1.3. Frequency

Some protective behaviors are easy to define in terms of topography and temporality but challenging to adopt in terms of frequency. For example, for handwashing to be effective, it needs to be compliant with the instructions of frequency, duration, thoroughness, and detergent use specified by research findings, see [24]. Frequent and diligent hand washing is one of the most cost-efficient behavioral measures, being listed as the first protective measure and public advice on the WHO’s website containing basic preventive measures [25]. Furthermore, it has been empirically addressed with increased attention by the scholarly community, e.g., [26].

#### 2.1.4. Effort

Effort is characterized as the vigor, intensity, energy, and expenditure required for a response to produce an outcome. During an outbreak in which there is no medical treatment available, change of habits may imply short and long-term costs. Interventions that aim to reduce the effort of protective behaviors and to change the magnitude of its consequences could retain the adherence to protective measures. For example, if there are no stores nearby or the price of hygiene products is high, there will be a significant cost to self-hygiene behavior [27].

### 2.2. The Antecedent-Behavior Component

The discriminative function of a class of stimuli is acquired during a specific learning history of signaling the relation between a behavioral class and its consequences [28]. If a behavior is followed by reinforcing consequences in the presence of certain stimuli but not in the presence of others, the former will gain a signaling function and increase the probability of similar responses in their presence. In the COVID-19 context, however, an individual’s learning history with specific discriminative events can represent a hindrance to their safety and the safety of others. For example, signaling the availability of social reinforcement in the presence of coworkers or classmates can favor physical proximity. To promote protection, it may be possible to add environmental cues signaling the possibility of infection—for example, through a novel avoidance history of disease contexts. In this way, the discriminative function of seeing a classmate at the playground or a coworker at the office (antecedents) is maintained for as long as there is a possibility of transmission (aversive consequences); conversely, the discriminative function is altered if the context changes.

During a pandemic, antecedents may also be powerful instruments of behavior change. In fact, some interventions to increase compliance with physical distancing include changing the salience of an antecedent by placing reminders at grocery stores [29] or in other public spaces [30]. Other interventions include the transfer of function with messages referring to physical distancing as a sign of respect and caring for others [31,32]. Furthermore, it is possible to implement antecedents that signal delayed consequences. These emphasize the link between physical contact and negative delayed outcomes both for individuals and at the level of public health [33,34]. In the remainder of this section, we illustrate these principles by offering an analysis of how they can be understood and applied.

#### 2.2.1. Salience of Antecedent Stimuli

The salience of antecedent stimuli can be increased through messages conveyed by news channels, international and governmental calls for action, public campaigns, and so on. Increasing the intensity of certain environmental events can favor the extent to which they are observed and responded to. Thus, this increases the likelihood of behaviors controlled by similar stimuli in previous contexts. For instance, stores may place salient floor and poster information signals with setting requirements for physical distancing [35], or they may provide audio warnings and hand sanitizer in strategic places [36]. However, this would be ineffective if individuals did not see the messages on the floor or hear the cues on the speakers. For this reason, a cluster of stimuli might be more effective. If antecedent events for protective behaviors are less salient than the opportunity for close social contact, the rates of the former are likely to be smaller—overshadowing; [37]. Even though increasing the salience of antecedent stimuli may increase compliance with protective behaviors, it may not guarantee their recurrence nor the necessary levels of engagement to lower R_0_ values—see [38]. It is necessary that the antecedents are salient and linked to the consequences of behavior, which are part of an individual’s learning history.

#### 2.2.2. Transfer of Function

Effective communication aimed at informing about the risk of contamination by physical contact can change the reinforcing value and the discriminative function of another person’s presence [39]. A motivating operation (MO) is a contextual event that significantly alters the effects of consequences on behavior (value-altering effect), and the evocative function of antecedents (behavior-altering effect) [40,41]. MOs can also alter the function of stimuli which influence behavior by decreasing the chances of future occurrences. This may alter the function of consequences from reinforcing to punishing and establish an antecedent to abolish behavior. Moreover, motivating operations have a behavior-altering effect through discriminative stimuli that were previously associated with a reinforcing or aversive event. Laraway et al. [40] noted that “the behavior-altering effects of MOs may depend on the presence of relevant discriminative stimuli” (p. 412)—see also [42]. Verbal stimuli are events that modulate such functions, mainly by facilitating the establishment of responding according to stimulus equivalence [43] when the function exerted by one stimulus is transferred to another that becomes part of the same equivalence class. In sum, effective communication could be a feasible way to promote the transfer of function by altering the motivational states and the role of antecedent events.

The discriminative function of events may change depending on how agencies describe the consequences of physical contact compared to the consequences of no physical contact. Formal and informal communication channels may be used to change the discriminative function of physical contact—for example, by pairing that behavior with a stimulus that elicits unpleasant responses, thus prompting a change in stimulus valence (i.e., evaluative conditioning) [44,45,46]. These behavioral processes can decrease the evocative power of another person’s presence and increase the averseness of their physical proximity while decreasing the probability of physical contact or proximity (i.e., an abolishing operation) [42,43,47]. Effective communication can also improve face mask wearing and other protective behaviors by describing and showing models of how it may be a sign of caring for and respecting the community. Thus, these and other favored environmental events may signal that protective behaviors are likely to be followed by social approval (consequence).

Establishing physical contact as a pre-aversive event may also be a feasible measure to increase the frequency of such behavior. As Sunstein [48] stated, “getting very close to other customers in a grocery store had, in many places, a clear social meaning: ‘I don’t care about your health.” (p. 3), which would hardly have been the case in the social context preceding the pandemic. Thus, it seems possible to foster physical distancing without necessarily enforcing it in terms of formal consequences (e.g., fines as punishment) beyond its function as a social norm [49]. Even without a history of direct association with unconditioned stimuli (i.e., each individual experiencing infection), environmental events can derive a signaling function of the high probability of aversive consequences, and thus decrease the occurrence of risk behaviors [50].

#### 2.2.3. Signal Delayed Consequences

During the early stages of a pandemic, low infection and mortality rates may signal delayed consequences of contracting the disease if protective behaviors are not adopted. The likelihood of engaging in these behaviors might be associated with the width of the temporal window during which reinforcers are integrated [51]. Individuals with shorter temporal windows tend to increase the value of more immediate reinforcers, while individuals with larger temporal windows may increase the value of more delayed reinforcers. Antecedent manipulations may extend individuals’ temporal windows and increase the value of delayed reinforcers. Interventions such as “episodic future thinking”, according to which one needs to vividly imagine and describe a realistic future event, [52,53] could be implemented on a large scale in an attempt to extend individuals’ temporal windows and increase the reinforcing value of adopting protective measures. Another example is to provide a description of the link between not adopting protective behaviors and the delayed consequences of an eminent pandemic outbreak [54]. Countries whose citizens’ behavior is under control of (i.e., have a history of correspondence between communication-contingency) warnings delivered by the scientific community and the government have an advantage in delivering effective messages about delayed outcomes.

### 2.3. The Behavior-Consequence Component

The effects of consequences on a behavior depend on several factors, including delay and probability—see [55], concurrent consequences [56,57], and whether an individual’s behavior produces positive and delayed consequences or avoids aversive and immediate consequences [58]. Many of the challenges in shifting behavior during the COVID-19 crisis are related to such paradoxes. In the next section, we describe the effects of consequences on protective behaviors. Specifically, we exemplify them by describing the effects of competing consequences, aversive versus reinforcing consequences, and the paradox of immediate versus delayed consequences.

#### 2.3.1. Competing Consequences

The allocation of behavior across activities may be understood from a paradigm of choice. According to this perspective, an organism’s engagement or avoidance in varied activities covaries with the frequency and magnitude of phylogenetically important events (PIEs) related to these activities. This leads to a permutation of preponderance of activities according to the relative distribution of PIEs, which are shaped throughout their natural selection history [56]. For example, socializing is a PIE, insofar as it is selected during our evolutionary history, but the specific forms through which individuals socialize are shaped throughout their individual learning history. Physical contact may provide frequent reinforcers with a greater magnitude when compared with socializing on online platforms. In the latter, the frequency of interpersonal interaction could be higher but with lower magnitude. Moreover, while physical contact is a PIE, the use of online tools to avoid physical contact is learned. In parallel, each form of behavior could produce aversive events, such as contracting the virus by physical contact, experiencing loneliness while distancing. As change can be hampered by these background learning histories, protective behaviors often imply refraining from activities that systematically vary together with both harmful (contracting the virus) and reinforcing (socializing) consequences. This conflict may be mitigated by enabling alternative behaviors and alternative sources of reinforcement.

It is essential that alternative activities are part of the repertoire of the practicing individual, that they are well designed, and easily accessible. Not everyone is able to work from home or socialize online; therefore, alternative activities must be available. Restraining activities without enabling alternative behaviors is ineffective and may produce collateral effects. For example, this may increase stress, aggressive behavior, boredom, depression, and the use of alcohol and other drugs as a function of deprivation of previous activities and their former reinforcing consequences [59,60,61,62,63].

#### 2.3.2. Punishing Versus Reinforcing Consequences

Among direct consequences, “social distancing helps limit contact with infected people and contaminated surfaces” [19]. From a functional standpoint, when physical distancing and other protective behaviors are maintained by preventing an aversive event from occurring (i.e., contamination, death, or peer’s judgment), they are negatively reinforced: that is, behavior increases in frequency after avoiding an aversive consequence. However, when there is a low adherence to protective behaviors at the population level and the rate of transmission is high despite individual compliance, the conditional relation between protective behaviors and the avoidance of aversive events seems broken from an individual standpoint (avoidance response) [64]. In turn, increased rates of transmission may create a context in which fewer individuals adopt protective behaviors, decreasing further the extent of compliance with rules that describe protective behavior in a deleterious spiral. This is a case of extinction of avoidance behavior, wherein negative emotional and aggressive responses have been extensively documented [65]. This scenario features unpredictable and uncontrollable aversive events [66], and is predictive of learned helplessness, apathy, and impulsivity, e.g., [67,68]. Some interventions introduce pre-aversive events in the form of law enforcement that include fines and imprisonment for non-compliers. For example, as of 27 March 2020, Singapore was one of several countries to threaten punishment for the violators of physical distancing norms and Argentina and Jordan for breaching quarantine rules [69]. In this way, immediate negative consequences are imposed for failing to comply to quarantine rules, with the severity varying according to national laws.

#### 2.3.3. Immediate Versus Delayed Consequences

Whereas physical contact is a powerful source of social reinforcement, the context of the pandemic poses different schedules of reinforcement with concurrent consequences that have different cost and reward delays. On one hand, proximal physical contact still produces immediate social reinforcers, however it also produces delayed aversive consequences (getting infected). On the other hand, there is an immediate averseness or cost to engage in protective behavioral measures (physical distancing), however it produces a delayed negative reinforcer (avoiding getting sick). Previous studies have shown that delayed consequences have a reduced value. “The tendency to devalue a reward when required to postpone its receipt” [70] is called delay discounting. Hyperbolic curves best represent this phenomenon, in which delayed consequences lose subjective value and individuals show higher chances of engaging in behaviors producing immediate rather than delayed consequences [71]. This imbalance implies a lower propensity to adopt protective behaviors. Impulsive choices intend a preference for the option that grants access to immediate reinforcers of lower magnitude (physical contact) and delayed aversive consequences (sickness), over the option that grants access to immediate aversive consequences (physical distance) and delayed reinforcers of greater magnitude (good health).

There are other variables that affect the rates of compliance with protective behaviors. For example, it is not possible to work remotely in several occupations, which may lead part of the population to economic stress and produce a negative shock on the collective’s income. Some studies suggested that negative income shock tends to increase the discount rate of delayed consequences. For example, Haushofer et al. [72] adopted an experimental setting in which the participants received “rich” endowments or “poor” endowments and found that negative income shock operated on the effects of discounting, wherein poverty increased discounting (i.e., increased impulsive choices as the variables were manipulated in a real effort task). Moreover, negative income shocks may also result in short-term choices, such as by reading narratives increasing discount rates, whether experimentally arranged or self-generated—similar to media content; [73,74]. Conditions underlying impulsive choices may lead to a variety of behaviors that aggravate the negative effects of the pandemic, directly and indirectly. They may decrease the adoption of protective behaviors and health habits that require self-control, and increase the occurrence of undesirable behaviors, such as the consumption of alcohol, drugs [75], and empty calorie food sources [76].

There are several interventions that show positive effects in fostering self-control [53,77]: for example, the use of commitment strategies to plan conditions for self-control at the individual and group level. These strategies create a contingency (or group contingencies, in the case of several agents) to favor compliance with self-controlled behavior. In organizational contexts, the government could offer fiscal and economic incentives to employers who commit to retain at least a portion of their employees rather than setting them on leave. Concerning self-hygiene, hand washing could be difficult to establish in communities without basic sanitation, but soap dispensers could be maintained by public and private initiatives at strategic points within a community as an emergency measure. Praise and approval for engaging in target behaviors provide immediate consequences that may be available in a covert form or through the social mediation of other individuals encompassing the agent.

Another way to improve self-control and social contact while maintaining interpersonal distance is to develop social or communitarian online networks, thus creating a collaborative decision-making process. Two studies suggested that collaborative decisions tend to decrease the discounting rate. Charlton et al. [78] observed that delayed outcomes were relatively more valuable (with flatter slopes and less discounting) when others are involved in the decision process. Additionally, the socially closer to one another the members of a group are, the lower discount rate they are likely to display. Similarly, Bixter et al. [79] observed that intertemporal preferences of individual group members shaped the decisions of a group (e.g., tending to present an average discounting rate of the individuals who “preceded” the formation of the group). Individuals’ choices are influenced by the experience of collaborative decision-making, as they are adjusted to the discounting rate of the group.

## 3. Putting Behavioral Principles Together: Example of Functional Analysis

A functional analysis consists of a systematic examination of the covariation between behavioral classes, antecedents, and consequences; it takes into account all the properties of each element that were described above. For example, analyzing physical distancing allows one to define how this behavioral class looks in terms of topography, temporality, frequency, effort, the context where the behavior takes place, identifying the antecedents in terms of salience level, discriminative function, and so on. It also identifies the consequences of behavior—how they are competing, their immediacy, whether they are reinforcing or aversive. It is important to note that the development and implementation of a behavioral vaccine must fit a functional analysis and be appropriate to (i.e., compatible with) the social context where it is applied. A functional analysis of targeted behaviors may reveal incomplete contingencies, in several ways. For example, when antecedents do not acquire discriminative function or when they are overshadowed by other stimuli present in the environment, their presence will not suffice to prompt behavior. Similarly, consequences can be delayed in time or competing, and so on. Hence, the absence of proper conditions may lead to low rates of adopting protective behaviors. At the population level, interventions that do not address any element of a contingency may lead to a lack of compliance with protective behaviors [80].

With a view to understanding the contingencies that may hinder behavioral change and the persistence of dysfunctional patterns at the individual level, we suggest a pragmatic effort to identify and rate their antecedent events and consequences. We suggest introducing an adapted version of the PIC/NIC Analysis [81] for rating the consequences of protective behaviors or any other target behavior. Originally applied to organizational behaviors within performance management, this analytic tool is used to identify and rate the occurrence and sustainability of pinpointed behavior. The initials of its acronym refer to potential functions, temporality, and probability of environmental consequences over analyzed behaviors. PIC refers to positive, immediate, and certain events, whereas NIC refers to negative, immediate, and certain. Although it is not a scientific method, it allows one to systematically analyze the often concurrently antecedent and consequences of behavior, from the perspective of the performer. It is particularly useful for understanding the obstacles to behavioral change by rating their consequences along three axes: (i) positive (P) or negative (N), (ii) immediate (I) or delayed (D), and (iii) certain (C) or uncertain (U). Throughout our analysis, we replace the positive and negative classification of consequences with reinforcing (R) and punitive (P) outcomes. In fact, positive and negative consequences are based on their value, whereas reinforcing and punitive outcomes are based on their function and suit better the objectivity of the analysis. Regarding antecedent events, we systematized their relational features according to their function on favoring behaviors’ occurrence: (i) salient (S) or faded (F); (ii) discriminative (D) or neutral (N). In addition, contextual events with the capacity of altering the reinforcing value of consequences should also be listed as motivating operations (MOs). Engaging in this analysis is a way of evaluating how consequences establish and maintain protective behaviors in the scenario of a pandemic. According to Lattal and Porritt [82], this allows decision makers to “examine and categorize the known environmental variables that act on the behavior of interest” (p. 33).

In the next section, we put forward brief and illustrative example of a functional analysis of physical distancing, which can be extended to other protective behaviors (e.g., hand washing, face mask wearing, isolation and quarantine, etc.). Moreover, we attempt a PIC/NIC analysis for explaining some of the consequences of physical distancing that are not only contingent (PIC, NIC), but also uncertain to a lesser degree (PIU), which are able to exert a stronger influence. Conversely, consequences that occur or may occur in the future are less powerful to maintain the target behavior. In terms of antecedents, conducting a functional analysis allows for identifying events from the behavioral setting that play different roles in favoring, inhibiting, or exerting no influence on the occurrence of the target behavior.

### Functional Analysis of Physical Distancing

Physical distancing is amongst the most effective behavioral measures against the further spread of COVID-19, and it aims to “minimize the risk of direct transmission of the virus via inhaled droplets” [83], p. 2. Before targeting how physical distancing may be shaped and maintained, it must be operationally defined. Guidelines have been issued and revised—for example, the recommended physical distance has gone from 1 m (or 3 feet) to 2 m (or 6 feet) [19], and back to 1 m in some countries—e.g., [84]. Moreover, assembling in crowds is prohibited in several countries (e.g., no more than 2 to 5 people, depending on local guidelines) and situations in which compliance with the minimum physical distancing cannot be met should be avoided whenever possible. This includes avoiding public transportation, purchasing groceries at off-peak hours, setting limits to how many customers may enter simultaneously a public premise, and canceling organized sports training and events. Table 1 depicts physical distancing and its antecedents in terms of discriminative or neutral, and salient or faded, and consequences in terms of reinforcing or punishing, immediate or delayed, and certain or uncertain.

Humans are ultra-social animals [85], and many of the reinforcers that shape and maintain our behavior involve physical contact. The presence of colleagues, friends, and family is often a salient antecedent, signaling (S, D) that greeting each other with a hug or handshake will be followed by immediate positive reactions such as smiles and a warm chat (R, I, and C). On the other hand, not engaging in physical contact may signal (D) indifference or coldness to others (P and I, C). In this case, peers are antecedents for influencing physical contact by both immediate reinforcers and punishers. Furthermore, the phenomenon of social discounting predicts that the interests of people who are socially more distant to the agent have “less value” than those who are closer [86]. This entails that the agent may physically distance from people they know, although failing to do the same in other circumstances [87,88]. Furthermore, the phenomenon of social discounting predicts that the interests of people who are socially more distant to the agent have “less value” than those who are closer [88]. This entails that the agent may physically distance from people they know, although failing to do the same in other circumstances [87,88]. Unless alternative antecedents aiming to change the discriminative function of antecedents and additional consequences are available, it is less likely for individuals to behave in a way that hinders infecting strangers or socially distant people.

Examples of interventions include media campaigns that emphasize pandemic health risks, and distance as a sign of respect and care for others. If well-implemented, they may change the discriminative function of peer’s presence, so it becomes an antecedent signaling (D) for interacting within a safe distance (i.e., 2 m). Media efforts can also transform the function of consequences, when interacting from a safe distance becomes a sign of caring, and physical contact a lack of respect. If such contingencies become part of a cultural practice, the presence of others will become an antecedent signing that physical distance leads to reinforcing, immediate, and certain consequences (R, I, and C), whereas physical contact will have a punishing, immediate, and certain consequence (P, I, and C).

Highlighting the possibility of getting sick by physical contact points out to punishing, yet delayed and uncertain consequences (P, D, and U). It is important, but it may not be sufficient to change the discriminative function represented by the presence of others. In this context, governmental agencies, parents, schools, non-governmental organizations, and the private sector may provide contextual-specific instructions describing other immediate and delayed consequences. Such instructions may inform the cumulative effect of noncompliance, the risk of infecting others even when symptoms are not present, and so on. Instructions are verbal stimuli aiming to alter the discriminative function of antecedents, motivational operations, and the value of reinforcement and punishment [39,89]. The efficacy of instructions will depend on the individual history of following instructions, but also on how instructions are phrased. They can target reinforcing, delayed, and uncertain consequences (R, D, and U), such as the effects of controlling local transmission rates in case of large-scale compliance, or it can focus on punishing, delayed, and uncertain consequences (P, D, and U)—for example, highlighting the spread of the virus.

Even when the presence of others acquires discriminative function for physical distancing, contextual modifications may increase compliance by altering the salience of antecedents. For example, signs may be added on grocery store floors to prompt the distance to be observed between customers, and benches in public spaces may be marked in a way that people avoid sitting next to each other (S). Similar to the function of a prompt, these comprise nudges which refer to environmental cues aimed at steering behavior without coercing it—see also [90].

Antecedents are environmental variables that covary with consequences, providing cues for the emission of behavior. Nevertheless, consequences ultimately select and maintain behavior, as well as the discriminative function of antecedents. Physical distancing may be maintained by several concurrent consequences: for a worker whose job depends on physical proximity, following guidelines of physical distancing will lead to a loss in income (P, I, and C), whereas caring out his/her work normally may lead to keeping, at least partially income (R, I, and C). Given that money is a generalized conditioned reinforcer (i.e., gives access to other reinforcers), the consequences for physical distancing may be weighted out by all reinforcers that can be exchanged for money. Thus, individuals whose income depends on physical proximity must have access to financial support, and/or the reinforcers that are accessible by physical proximity. This includes cash transfer programs and the provision of other reinforcers (e.g., support for basic needs, such as rent and food). When possible, initiatives must provide the means for individuals to engage in work activities, while observing physical distancing. Otherwise, interventions that address behavioral change exclusively through the manipulation of antecedent events might only have moderate and temporary effects.

To overcome some of the gaps in the behavior-consequence component of a behavioral contingency, controlling agencies could override delayed reinforcers and immediate aversive consequences of physical distancing by assigning immediate benefits [91]. For example, companies, public spaces, and other agglomeration environments (e.g., public transportation) that manage and deploy measures for safeguarding physical distancing, could receive tax discounts, media recognition, and awards at events in specific sectors of commerce (R, I, and C). Thus, they could establish a concurrent reinforcing contingency.

Physical distancing can cause social isolation, as proximity starts to produce aversive consequences, while distancing leads to reinforcing events. The greater the isolation, the more likely the individual is to experience an extinction procedure and process; hence, unpleasant emotional responses. Reducing the costs of alternative responses (substitute events) may comprise an alternative. The consequences of physical distancing may have transformed through the mediation of internet-based technology. The probability of social experience without contact could increase if the costs of internet, computers and all devices were reduced [92]. Besides, individuals can gain access to other activities that could also be enjoyed. For instance, attending a streamed online concert permits experiencing a similar (yet attenuated) music enjoyment rather than attending a live concert in person.

These examples of functional analysis adapted from PIC/NIC analysis point to some gaps challenging compliance to physical distancing. Next section advances a framework resting on culturo-behavior science for planning interventions at population level. We argued that effective interventions should incorporate a well-established functional analysis in order to cluster the three elements. It is important to consider that incomplete contingencies may be inefficient to promote physical distancing or any other behavior over time. Nevertheless, during a pandemic, countermeasures should be subsidized and not taxed by their social meanings [48]. Once the environmental variables (antecedents and consequences) are known, competent agencies should organize actions to ensure behavioral change on a large scale, in a coordinated fashion. Controlling agencies are composed of the interchanged behavior of more than one individual and have the power to control contingencies on a large scale by adding, removing, or restricting access to environmental events. They should form a dynamic system, a national or culturo-behavioral immunological system to combat the pandemic, which we describe in the following section.

## 4. Developing a System Response to COVID-19 and Preparedness for Future Outbreaks

The widespread administration of pharmacological vaccines not only affects immunized individuals, but it also has an indirect effect at the population level: this is termed herd protection—or herd immunity; see [93]. While the consequences of not participating in vaccination programs are delayed and probabilistic, insofar as it may be possible to contract a disease, there is an immediate response cost to participating (e.g., time, effort, and possible mild collateral effects). Moreover, the cost of engaging in a vaccination program weighed against the probability and delay to be infected is affected by the number of committed individuals. If enough people are immunized, the individual cost of vaccination does not change, whereas the probability of infection is both decreased and delayed. It is possible to observe similar paradoxes in the case of a behavioral vaccine.

Although cooperating at the level of small groups is part of our evolutionary history, behaving towards group-relevant outcomes depends on constrains for self-serving practices. These within-group dynamics aim at preventing undesirable outcomes of conflicting contingencies between individual interest and group common goals. Outcomes that are relevant for society can be even less prominent and, therefore, suffer higher levels of discounting. Undesirable outcomes at the societal level can be prevented by implementing interventions that deliberately manipulate variation and selection processes, taking into account the inherited capability to learn from environmental consequences (i.e., the behavior-consequence component) and inherited behavioral patterns (phylogenesis) [94]. Increasing the rate of compliance with protective behavior ought to take these variables into consideration when designing antecedents and consequences that can connect individuals’ behaviors to the delayed and probabilistic effects of non-compliance.

Whereas the behavior of each member in a population-wide response is influenced by its interaction with the environmental variables (antecedents and consequences), the cumulative effect of the cultural practice reflects the population’s cumulative behaviors at large. As long as the only currently effective resource to control the COVID-19 pandemic is the population’s adherence to the advice of the health authorities, the R_0_ and CFR will vary according to each nation’s completion of three-term contingencies. Thus, a functional analysis of contingencies and practices should consider both individual behavior and cumulative effects of those practices [52]. Conversely, simple gaps can determine the failure of large-scale strategies that aim at promoting novel practices.

**Figure 2:**
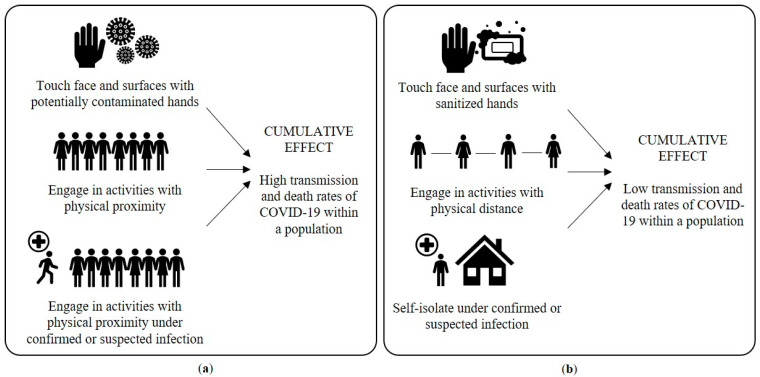
(**a**) Defective macrocontingency comprised of behaviors that increase the spread of COVID-19; (**b**) protective macrocontingency to contain the spread of COVID-19.

Cultural practices are shared behavioral patterns that, when accumulated, can positively and negatively influence the outcomes of a social issue, such as the rate of a virus spread. In the context of the COVID-19 pandemic, they can be measured as rates of engagement with protective behaviors advised by health authorities, whereas their cumulative effect is measured as infection and death rates, as well as social and economic indicators [95]. Macrocontingency is an approach used to address the cumulative effect generated by several individual behaviors, which in sum can have a social impact [96]. Figure 2 depicts the relationship between non-recommended (a) and recommended (b) behaviors, and their cumulative effect when presented at large scale within a macrocontingency.

A timely adoption of protective behavioral practices has been shown to be an effective measure against the rapid rise of infection rates of the COVID-19 in several countries—e.g., [97]. We argue that linking and embedding them into cultural practices could possibly prevent larger social and economic effects of the pandemic, such as gender differences [98] or cultural complexities and biases [99]. Others have called for integrating findings from behavioral economics and infectious disease epidemiology for mitigating the pandemic, arguing that interventions should aim at changing social norms and spread further throughout the network [100]. Providing appropriate antecedents and consequences to complete potential gaps in the three-term contingency (antecedent-behavior-consequence) of individual behavior plays an important role in shifting behavior and cultural practices, and therefore preventing outbreaks from reaching uncontrollable levels. Thus, change can be promoted by manipulating variables that favor safer variation from previous behavioral patterns, selection of valuable practices for group-maintenance, and their replication at the population level. It is possible to achieve this shift by synchronizing the action of different agencies, including the media, the educational and health care systems, scientific and professional associations, and others—e.g., [101]. This implies envisioning the compound effects of reinforcing individual behavior in the short-term and the cultural selection of their combined effects.

In the next section, we turn to community-level analysis and discuss how behavioral interventions that are effective at the individual level can be extended to the level of groups and social systems.

### 4.1. From Macrocontingencies to Metacontingencies

In order to reach stable protection levels, the coordinated agency action towards shifting and maintaining protective practices must be sensitive to the different stages of a pandemic. For example, when epidemiological monitoring agencies detect a pathogen that can potentially lead to exponential spread, governmental executive agencies should promote context and consequences to stop it in a timely fashion. Next, the media and other societal organizations have the task of spreading this information. In such a network of coordinated actions, each agency’s initiative becomes an antecedent or consequent event for the other agency’s initiative, which is similar to the form of interlocking contingencies. Thus, we now extend the unit of analysis from individual contingencies to group contingencies (i.e., consequences contingent on the joint behavior of individuals in a group) to inform policymaking.

Within the context of an increase in the number of COVID-19 cases, the punctual provision of antecedents and consequences to favor protective behaviors can be efficient in promoting individuals’ compliance. However, as the normalization phase approaches and the environment is less informative of the risks of infection, the maintenance of protective measures in a large-scale is less likely. Together with a phase of deceleration, population engagement might diminish, resulting in loosening compliance with hand washing, physical distancing, quarantine, and other recommended or mandatory practices. To prevent a forthcoming stronger wave or any future outbreaks, cultures need to incorporate the recommended behaviors since the initial signs of contagion. Although cultural practices emerge without the need for designs for intentional change [102], advancements in the culturo-behavior approach can inform policies that favor a faster and more effective development of new practices on a large-scale [103]. Thus, we discuss how a higher level of organized complexity requires the analysis of the interdependent behaviors of several members of the group, be it a community or society. The correct analysis of these behaviors is key to establishing appropriate, effective, and sustainable cultural practices, as these necessarily involve the coordination of individual efforts.

Behavioral patterns within a population are favored by physical and social similarities in the context—see [104]. However, the variables affecting behavior at a cultural level can overlap with a large variety of other stimuli that were important throughout the individual’s learning and genetic background. Therefore, environmental cues that influenced behavior in the past may regain their controlling prevalent effect as soon as routines are re-established, unless efforts are taken to strengthen community engagement for an extended period. A promising path involves the development of interconnected networks of behavioral contingencies, rather than interventions uniquely at the individual level.

### 4.2. Metacontingencies of Support

The conditional relation between coordinated efforts of individuals (or organizations) towards an aggregate product and a cultural consequence is termed a metacontingency [96]. This concept encompasses processes through which interlocked behavioral contingencies (IBCs) are selected by the environment as one unit, together with the aggregated products from agents’ interactions. Selection occurs at the cultural level, which means that the recurrence of a network of contingencies is dependent on cultural consequences. This functional relationship has been demonstrated in basic research [105], and has been identified in laws, social policies, and cultural phenomena [95,106,107].

Exploring the relevant elements in the selection and maintenance of coordination towards the “common good” can help agents from public or private spheres to contribute with the promotion of behavioral changes at the population level. In the midst of the COVID-19 crisis, favoring preparedness involves a deliberate cultural design addressed to enhance the rapid engagement in protective behaviors. Figure 2 depicts a general example of coordinated efforts from different agents towards the promotion of a new behavioral pattern with protective effects at the population level. The illustration explores the intended change in defective behaviors that accumulate a high transmission rate in the face of the first signs of a respiratory infection into protective behavioral patterns—from maintaining activities with physical proximity to self-isolating.

This example comprises (1) a defective cultural practice as a societal problem due to its cumulative effects; (2) coordinated actions from different agents or agencies with the common goal of reducing the societal problem through the promotion of a new behavioral pattern; (3) the alternative behavioral pattern that can protect a community; and (4) selective consequences contingent on the targeted protective cultural practice. Government, science, media, schools, services, and non-governmental organizations are illustrated as examples of possible agents in a coordinated response towards fostering change in behavioral patterns at the population level. The agents, their roles, and the communication patterns within a system can vary according to the social issue, available resources, local practices, organizational structures, and so on, affecting their aggregate outcomes and their selection by cultural consequences. In the following paragraphs, we guide the reader through important elements and actions translated into general steps in which agents or agencies could engage to favor a rapid and successful implementation of cross-sector action to contain the spread of the virus. Special attention is given to strengthening the coordination between agents, which is key to providing a cohesive environment that can design and sustain novel cultural practices.

#### 4.2.1. Define a Common Behavioral Goal

The establishment of successful interaction networks that can promote a permanent large-scale behavioral change requires that agents clearly define the aspect of the societal problem towards which they want to direct their combined action and affect. For example, the aim of “mitigating the COVID-19 crisis” would be partially informative regarding the practical steps in which each actor can engage. To gain precision, agents should identify which behavior they can impact by the sum of their efforts and doing so on a large-scale. For instance, stating the aim as “increasing the occurrence of self-isolating under signs of suspected infection” renders it more feasible to pursue, monitor, and assess.

**Figure 3:**
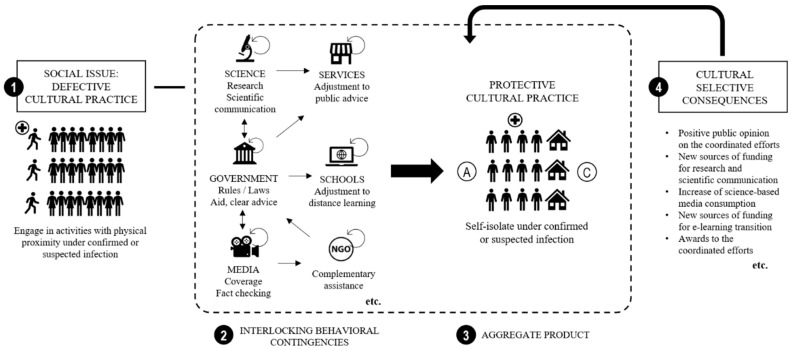
Metacontingency of support illustrating how defective practices (**1**) may require agencies’ coordination (**2**) in order to enhance population engagement in protective behaviors (**3**). Note: A = antecedent events; C = consequences.

In the case of any future outbreak, protective behaviors might not be as clearly recommendable as during the COVID-19 crisis. We suggest conducting functional analyses of the defective cultural practices that jeopardize a community’s resilience and deriving alternative behaviors that could considerably improve health indicators in the same context. For example, this can be seen in elements 1 and 3 in Figure 3, where self-isolation is proposed as the alternative to physical proximity at signs of infection. In some cases, it may be needed to develop new behaviors; in others, it may suffice to learn new topographies of old behaviors (e.g., handshaking).

#### 4.2.2. Identify Essential Actions Towards Target Behavior

While setting arrangements to favor new behaviors or topographies is important, so is ensuring the conditions that promote their recurrence. Agents can work on antecedent conditions that set the occasion for the emergence of new topographies or alternative behaviors, and on access to reinforcers to establish and strengthen them. This step can be unfolded into two lines of action: (i) expanding the class of responses of “defective behaviors” to include safe topographies, and (ii) establishing new behaviors. The former includes presenting new antecedent conditions that can set the occasion for the emergence of new or less frequent responses with equivalent functions—for example, providing free internet access to replace traditional classes with distance learning strategies. Establishing alternative behaviors requires the arrangement of both new antecedent conditions and new reinforcers. Self-isolating at mild signs of respiratory infection is not a common practice in every culture, but it should be established in the future. This way, the risk of spreading the pathogen might not suffice to control one’s compliance with health advice and additional environmental stimuli might be necessary. The concurrent consequences of attending a work meeting, a social gathering, or any other outdoor activity must be balanced by planned contextual events and short-term consequences.

To identify the essential actions to be taken, agents must map the potential gaps in the control exerted by the available antecedents and consequences in the context of the population, as exemplified in the functional analysis depicted in Table 1, taking into consideration the population’s background and repertoire. The expected aggregate product of the coordinated efforts between agents is illustrated in Figure 3 as the reoccurrence of a high adherence to self-isolation under confirmed or suspected infection.

Although physical distancing is enforced to limit any contact among individuals, it is still possible to engage in common activities and, thus, to gain access to their reinforcers while physically apart. For example, people can still engage in video chats and phone calls, hike in small groups while maintaining the recommended physical distance between one another, arrange a movie night within the household or online with friends, or work out at home while in quarantine or self-isolation. It is important that the agents and agencies involved in a coordinated response consider that new illnesses might also derive from the adoption of new topographies, such as addictions associated with prolonged exposure to screen time. In this case, the development of varied alternative topographies might be important while encouraging a reasonable balance across daily activities.

#### 4.2.3. Map Essential Roles and Identify Promising Agents

Every essential action requires identifying roles with proper tools to perform them: these include executive, legislative, and judiciary branches; universities and research institutes; media; non-governmental organizations; representatives from specific populations; and others. For instance, possible roles and agents are illustrated in Figure 3 under element 2, interlocking behavioral contingencies (IBCs). The efficient delivery of environmental stimuli that both favor and strengthen behaviors at a large-scale level is affected by agencies’ resources, channels of communication, and access to those in need of interventions [108]. In this sense, it is preferable that an agency’s work is supported by a framework that ensures the availability of resources and continued actions. The possibility of the recurrence and transmission of practices is an essential aspect to be considered, regardless of the role played by a specific agent at whichever point in time.

In terms of access, effective measures can be taken by non-traditional agents. One example from the HIV epidemic was the program Live Better Knowing from the Ministry of Health of Brazil [109] in a combined effort with civil society organizations. Taking into account the stigmatized individuals who avoided seeking health care, representatives of the populations that were most affected by HIV were trained to perform oral tests to their peers. Through agents with privileged access to the target individuals, this strategy provided a clearer picture of the disease prevalence and facilitated governmental efforts of combined prevention. In the context of COVID-19, one of the most populous slums of São Paulo trained 240 of its residents as first responders and set up local emergency bases [110] thanks to the joint effort of unions, residents, the local fire department, and volunteers. Similar patterns were found by Ardila Sánchez et al. [95] in Puerto Rico while recovering from a natural disaster, wherein local agents worked collectively to provide solutions to the problems faced by the communities.

In terms of resources, if the interests of governmental leaders towards protecting power perpetuation overlaps societal needs, companies, citizens, and non-profit organizations have an even more crucial role during emergency situations [95]. Some agents may have financial, material, or conceptual resources that can favor large-scale behavioral change, but they may not have the means to make them accessible to the target individuals. Hence, their support must be combined with efforts from agents with delivery capacity. Concerning channels of communication, their relevance relies on how they can contribute to efficient coordination between agents in crisis scenarios. Delays, contradictory messages, and a lack of training can impair the ability of the system to promote a quick response for protecting the population [111].

#### 4.2.4. Connect and Coordinate Actions

After identifying the collaborating actors, the next step is to connect them, as illustrated in Figure 3 by the arrows between the agents whose behaviors are part of a coordinated response. Antecedents and consequences need be presented in a synchronous manner to effectively establish the target behavior. Uncoordinated actions might result in the presentation of competing contingencies [112]. Public health agencies might recommend physical distancing without necessarily collaborating with other sectors responsible for education, labor, and technology. In this case, the modification of individual repertoires is unlikely. Paychecks are delivered to workers performing inside an organization; grades are assigned by a teacher who attends to a student taking a test at school; psychiatric services are delivered by physicians to patients in medical offices. In all these situations, engaging in activities with physical proximity is immediately reinforced. People may face a conflicting situation without a strong commitment from agencies to change their guidelines and present antecedent conditions to include the possibility of engaging in the same activities with physical distance. While physical distancing avoids harmful health outcomes, physical proximity grants access to other immediate social outcomes, such as wages, grades, or treatment.

#### 4.2.5. Highlight Outcomes from Coordination

The selection of cultural practices that are beneficial for society should depend exclusively on the status of their cumulative effect. In the context of the COVID-19, this is illustrated in Figure 2 by the low transmission and death rates that can result from the set of novel protective cultural practices. When a system response is designed to and aims at promoting high adherence to a protective behavior, the aggregate product from the combined efforts by different agents corresponds to the accumulated effects of the novel practice. In this sense, outcomes on relevant health indicators from coordination should be highlighted to enable a feedback loop on their underlying actions. The provision of reliable information contingent on the achieved aggregate products facilitates the evaluation of the effectiveness, relevance, and sustainability of the actions. Moreover, it creates an opportunity for continued collaboration towards the intervention plan, improving strategies or adjusting the route as necessary.

Actors need be able to verify the association between relevant health indicators (or their cumulative effect), such as changes in infection and death rates, and individual behavior (e.g., hand washing in public spaces and physical proximity). Hence, a monitoring and evaluation system could be implemented for reporting infection and death cases from local to central public health agencies in the fastest possible way so that the data inform the aggregate products of coordinated action. Real-time reporting using surveillance technologies including mobile data and tracking could be used as an alternative, although this approach brings ethical limitations that should be considered and overcome. In highly complex networks, the contribution of each actor in controlling infection and death rates might become clearer through intermediate and unbundled indicators. They should be more prone to repeat relevant behaviors in a coordinated fashion by having access to information on how their action increased the protection and safeguard among assisted communities.

Publicly accessible data of the social benefits achieved by a coordinated system would be not only a promising reinforcer to the behaviors of each actor involved but could also set cues for the population, endorsing their joint efforts. The aim is to facilitate the generation of cultural consequences that can select the combined efforts supporting the beneficial cultural practices and, therefore, their maintenance.

#### 4.2.6. Cultural Consequences

The last aspect of a metacontingency to which we draw attention is its receiving system, as illustrated by item 4 in Figure 3. The environmental events contingent on coordinated actions and their aggregate products can favor or hinder their recurrence. In the case of designed interventions, the timely visibility of the socially relevant aggregate products of a system response might be pivotal to increase its recurrence. While the coordinated efforts from agents are necessary for the provision of environmental influence on protective behaviors, the supporting agencies also need to have their initiatives strengthened. One might create a context for cultural selection processes by favoring public endorsement. The examples explored in Figure 3 go from cultural consequences that acknowledge the combined efforts of different agents as a whole to environmental events contingent to sub-systems in the web of interactions. Whilst citizens’ endorsement of health workers’ action in the front line of COVID-19 illustrates the increase of positive public opinion on coordinated efforts of involved agents, anti-lockdown protests exemplify how the receiving system can also respond in a way that adds obstacles to the maintenance of coordinated interventions.

In the case of community preparedness for the rapid installment of protective behaviors during future outbreaks, the more interconnected the supporting agencies’ behavioral contingencies are, the more efficient they can be at detecting early signs of future infection waves. Thus, a cultural design can benefit from anticipating possible responses from the social environment, and from planning external visibility that can favor external demand which functions as a selective environmental consequence.

## 5. Conclusions

This work features the possible underlying principles of a COVID-19 behavioral vaccine for a sustainable cultural change. It provides an analysis of some of the behavioral variables for positive change during the outbreak of the current pandemic. We suggest that the present systematization of conceptual tools can favorably impact society in at least two ways. First, we put forward a framework for designing strategies to promote behavioral change based on the identified gaps in the three-term contingency of the target behaviors in each context. Then, we scaled up individual-level analysis to inform community-level policymaking. Second, we state that behavioral and cultural strategies to promote preventive practices are crucial to the effectiveness and sustainability of interventions. These should be functionally analyzed in terms of behavioral contingencies, scaled to macrocontingency analysis, and next metacontingencies of support should be designed to assure that a system response takes place and that cultural consequences are sustained. Specifically, the combined and mutually interdependent efforts of both agents and agencies are called to manage and contain the spread of the virus.

Developing and impoverished countries face additional challenges in terms of their economic hardship [113], which limits the long-term maintenance of lockdowns and other restraint measures. Notwithstanding this, quarantine and physical distancing have limited feasibility for informal workers and other vulnerable populations whose daily earnings are necessary to meet their basic needs. Thus, disproportionate social protection may exacerbate inequalities and expose population segments differently to the risks of infection due to the further obstacles present in underprivileged communities to adopting and maintaining the advised practices. We suggest that cohesive and coordinated policymaking action for delivering environmental events that favor adherence to protective behaviors at a large scale is key to reducing the length and severity of future outbreaks. Consequently, unnecessary health, social, and economic fragilities at the individual and community levels can be avoided. For example, Williams and Kayaoglu [114] suggested further policy responses beyond the effects of the COVID-19 on the undeclared economy and Gros et al. [3] modelled the economic recuperation with the implementation of behavioral measures by balancing public health and the economic costs of a lockdown.

Lastly, it is noteworthy that the development and implementation of a behavioral vaccine must be appropriate and compatible with the social context where it is applied. Therefore, any intervention proposition must involve ethical respect for the community’s practices, and it must qualitatively probe their needs and objectives [115,116,117,118]. It is of the utmost importance that a functional analysis and the following behavioral interventions are transparent and follow agreed-upon guidelines. They should be in the interests of the public, community, and laws and follow scientifically proven measures.

## Figures and Tables

**Table 1:** Functional analysis of physical distancing with ratings inspired by the PIC/NIC Analysis tool and stimulus control.

Antecedents	S/F	D/N	Response	Consequences	R/P	I/D	C/U
Presence of other	S	D	Physical distancing (2 m or 6 feet apart from one another)	Income loss (when physical contact is necessary to work)	P	I	C
Poster on the risks of physical proximity	S	D		Avoid getting sick	R	D	U
Signals (marks on the floor)	S	D		Avoid infecting others	R	D	U
Instructions		D		Social recognition	R	I	U
				Avoid judgment	P	I	U
				Control of local transmission rate	R	D	U

MOs: R_0_ of the pandemic; government advice; costs of the substitutes. Note: PIC/NIC = positive, immediate and certain/negative, immediate and certain; S/F = salient/faded; D/N = discriminative/neutral; R/P = reinforcing/punitive; I/D = immediate/delayed; C/U = certain/uncertain; MOs = motivating operations; R_0_ = reproduction rate.
